# Supplementary material for: Co-Developing an Antibiotic Stewardship Tool for Dentistry: Shared Decision-Making for Adults with Toothache or Infection
Source: Antibiotics (Basel). 2021 Nov 4;10(11):1345. doi: 10.3390/antibiotics10111345 (PMC8615064; doi:10.3390/antibiotics10111345)
Supplement: Supplementary file 1 [file antibiotics-10-01345-s001.zip › antibiotics-1445932-supplementary.pdf]

Supplementary material

Table S1: Candidate Behaviour Change Techniques shortlisted for the ABSCESS intervention by application of the APEASE criteria (Affordability, Practicability, Effectiveness, Acceptability, Side effects/Safety, Equality. Source HBCP, 2018

TDF Domain	Factor	BCT with confirmed links to the literature, expert consensus or triangulation	Application of the APEASE criteria	Fit with shared decision-making intervention
Beliefs about consequences	Antibiotic beliefs	5.1 Information about health consequences<br>5.2 Salience of consequences<br>5.3 Information about social & environmental consequences<br>5.5 Anticipated regret<br>5.6 Information about emotional consequences	5.1 Yes<br>5.2 Yes<br>5.3 Yes<br>5.5 Yes<br>5.6 Yes	5.1 Information about health consequences<br>5.2 Salience of consequences
Environmental context/resources	Competing demands	1.2 Problem solving<br>3.2 Social support (practical)<br>7.1 Prompts/cues<br>7.5 Remove aversive stimulus<br>11.3 Conserving mental resources<br>12.1 Restructuring the physical environment<br>12.2 Restructuring the social environment<br>12.3 Avoiding/reducing exposure to cues for the behaviour<br>12.5 Adding objects to the environment	1.2 Not affordable<br>3.2 Not practicable<br>7.1 Yes<br>7.5 Not practicable<br>11.3 Yes<br>12.1 Not practicable<br>12.2 Yes<br>12.3 Yes<br>12.5 Yes	7.1 Prompts/cues<br>12.2 Restructuring the social environment<br>12.5 Adding objects to the environment
Goals	Fix the problem	1.1 Goal setting (behaviour)<br>1.3 Goal setting (outcome)<br>1.5 Review behavioural goal(s)	1.1 Yes<br>1.3 Not practicable yet<br>1.5 Not practicable<br>1.6 Not practicable yet	1.1 Goal setting (behaviour)

		1.6 Discrepancy between current behaviour and goal<br>1.7 Review outcome goal(s)<br>1.8 Behavioural contract<br>8.7 Graded tasks<br>10.2 Material reward (behaviour)	1.7 Not practicable yet<br>1.8 Not practicable<br>8.7 Not practicable<br>10.2 Unaffordable	
Social influences	Patient influence<br>Peers & colleagues	2.1 Monitoring of behaviour by others without feedback<br>3.1 Social support (unspecified)<br>3.2 Social support (practical)<br>6.2 Social comparison<br>6.3 Information about others' approval<br>10.4 Social reward<br>12.2 Restructuring the social environment	2.1 Not practicable yet<br>3.1 Not practicable<br>3.2 Not practicable<br>6.2 Not practicable yet<br>6.3 Yes<br>10.4 Yes<br>12.2 Yes	12.2 Restructuring the social environment
Skill	Patient management	1.2 Problem solving<br>4.1 Instruction on how to perform the behaviour<br>6.1 Demonstration of the behaviour<br>8.1 Behavioural practice/rehearsal<br>8.6 Generalisation of target behaviour<br>8.7 Graded tasks<br>10.9 Self reward	1.2 Not affordable at scale<br>4.1 Yes<br>6.1 Not practicable<br>8.1 Yes<br>8.6 Not effective<br>8.7 Not practicable<br>10.9 Yes	4.1 Instruction on how to perform the behaviour
Beliefs about capabilities	Planning & consent<br>Procedure possible	1.1 Goal setting behaviour<br>1.2 Problem solving<br>2.6 Biofeedback<br>4.1 Instruction on how to perform the behaviour	1.1 Yes<br>1.2 Not affordable<br>2.6 Not effective<br>4.1 Yes	4.1 Instruction on how to perform the behaviour

		6.1 Demonstration of the behaviour<br>8.1 Behavioural practice/rehearsal<br>8.7 Graded task<br>10.4 Social reward<br>11.2 Reduce negative emotions<br>15.1 Verbal persuasion<br>15.3 Focus on past success<br>15.4 Self talk	6.1 Not practicable<br>8.1 Yes<br>8.7 Not practicable<br>10.4 Not practicable<br>11.2 Yes<br>15.1 Yes<br>15.3 Risk of side effect<br>15.4 Not acceptable	15.1 Verbal persuasion
Professional role & identity	Professional role	3.1 Social support (unspecified)<br>6.2 Social comparison<br>9.1 Credible source<br>13.5 Identity associated with changed behaviour	3.1 Not practicable yet<br>6.2 Not practicable yet<br>9.1 Yes<br>13.5 Yes	9.1 Credible source

Figure S1: Original draft of the worksheet used in the think aloud study.

7. When should I get additional help?

Call your dental practice or NHS111 if your symptoms do not improve within 48 hours.

CALL 111
when it's less urgent than 999

Go to A&E if you have

Signs of serious infection:
- Swelling is closing your eye or affecting your lip
- Swallowing or breathing is difficult
- Your temperature is over 38°C or under 36°C
- Your heart is racing
- You feel confused

OR

Signs of a serious reaction to antibiotics:
- Swelling of your lips, tongue or throat
- Trouble breathing
- Feeling faint or dizzy
- Watery diarrhoea with blood or pus in it.

These may be life threatening – seek medical advice

Notes:

Before your appointment:
You may wish to write down your symptoms and concerns to share with the dentist during your appointment.

During or after your appointment:
You may wish to write down things to remember

For more information about urgent dental problems:
www.nhs.uk/conditions/toothache/

Ref: DREC….. v1 dated 03 December 2018

Preparing to Fix Your Toothache

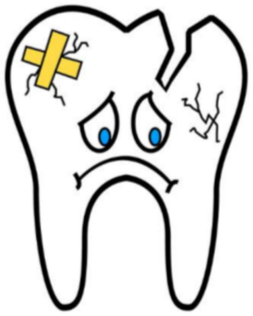

1. What is this leaflet for?

This information leaflet is to help you prepare for your urgent dental appointment.

It explains some of the most common urgent dental problems and treatments for them.

You and the dentist can use it to discuss options for fixing your problem today.

There is space on the back to keep notes for yourself.

2. Do I need antibiotics?

To fix dental problems quickly and stop them coming back, a dental procedure is usually needed.

Antibiotics may sometimes be required as well, but not instead of a dental procedure.

Signs of infection which might need an antibiotic include:

- Swelling visible on your face or neck
- High body temperature
- Difficulty opening your mouth

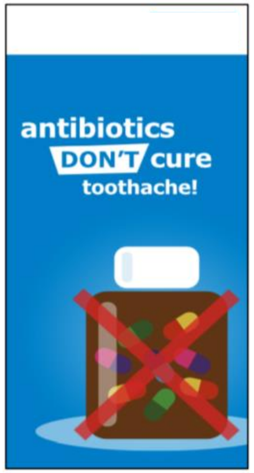

For more information about antibiotics in dentistry
www.gov.uk/guidance/
dental-antimicrobial-stewardship-toolkit

3. What is causing my dental problem(s)? (Tick box(es))
After asking you questions and doing tests to work out what is causing your problem, the dentist will select a cause below.

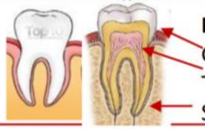

Healthy tooth
Gums
Tooth's nerve
Supporting bone

☐ 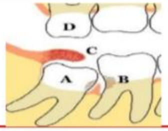

Wisdom tooth problem
Gum around the tooth hurts.
Tooth's nerve is fine.

☐ 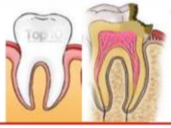

Toothache
Nerve in the tooth hurts, usually due to decay or a crack. The tooth may be dying.

☐ 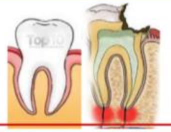

Dead tooth
Nerve in the tooth is dead.
Nerves in the bone may hurt.
An abscess may start forming.

☐ 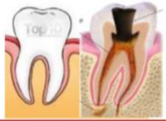

Dental infection
Nerve in the tooth is dead.
Nerves in the bone may hurt.
Pus collects around the tooth

☐ 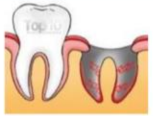

Dry socket
After a tooth has been removed
Nerves in the bone may hurt
from slow healing not infection

4. What procedure(s) may fix my problem(s)?
Your dentist will select and explain which of these options may fix your problem today. (Tick box(es))

☐ **Cleaning under the gums or in the dry socket**

☐ **Normal filling**

☐ **Nerve/pus removal and temporary dressing**

☐ **Tooth removal**

☐ **Something else**________________________

You then decide what you want to have done.

Remember to ask about any extra treatment you might require to stop the problem coming back.

Your dentist may use this diagram to explain which tooth needs treatment and any issues to consider.

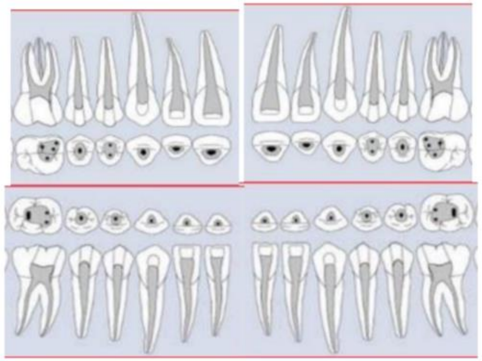

Figure S2: Participant information sheet used in the think aloud study.

Ref: DREC101218/WT/267 v3 dated 18 December 2018

UNIVERSITY OF LEEDS

FACULTY OF MEDICINE & HEALTH
SCHOOL OF DENTISTRY

PARTICIPANT INFORMATION SHEET

APTiTUDE/TRUCE Think Aloud Study (ATTAS)

Thank you for your continued support of the TRUCE Study. For the next stage, I would like to invite you to take part in a feedback session with me at the University of Leeds or, if you prefer, by telephone.

What is the purpose of this study?

In this feedback session, I am interested in what you think about when you read a prototype communication tool for use by dentists and patients during urgent dental appointments. Your feedback will help improve the tool before it is tested more widely in dental practices and clinics.

Who is doing the study?

Wendy Thompson is a dentist and PhD research student; this study will form part of her research. Professor Gail Douglas and Professor Sue Pavitt are Wendy's PhD supervisors.

Who is being asked to participate?

As one of the APTiTUDE/TRUCE Multi-Disciplinary Group advisors, you are being asked to participate.

What will be involved if I take part in this study?

Consent: You will be invited to sign a consent form confirming that you understand what the 'think aloud' study is about and that you are happy to provide feedback about the prototype communication tool.

Audio-recordings: Your feedback session will be recorded using a University of Leeds digital voice recorder which has data encryption. After being transcribed (typed up), the audio-recording will be deleted.

Feedback: The feedback session should take no more than 30 minutes and at a time which suits you best. As I am interested in your first impressions of the prototype communication tool, you will see it for the first time at the feedback session There are no right or wrong answers. The sorts of things you might 'think aloud' about are:

1. Looking at the leaflet cover, what did you think it was going to be about?
2. How did it make you feel when you first saw it?
3. For each section: What did the section tell you? Why do you think it is here?
4. What are your overall thoughts on the sheet?
5. How can it be improved?

6. What will stick in your mind from this leaflet?
7. How easy or difficult was it to read/ understand?
8. How would you describe it to your friends, family or colleagues?

What are the advantages and disadvantages or risks of taking part?

There are no advantages, disadvantages or risks of taking part. Your time and any travel costs for participating will be reimbursed.

Can I withdraw from the study at any time?

You are free to withdraw your consent at any time up until your data is no longer individually identifiable; this will be not sooner than one week after your interview. You do not need to give a reason for withdrawing. If you decide to withdraw, please let me know using the contact details below.

Will the information obtained in the study be confidential?

All information collected during the course of this research will be kept securely within the University of Leeds and its systems. Audio-recordings will be destroyed once transcribed. Anonymised data will be retained in the Research Data Leeds Repository for 5 years after the end of the study. This will allow it to be shared with other researchers who may use it to support other research and/or publications. Confidentiality will be respected, subject to legal constraints and professional guidelines.

Who has reviewed this study?

Approval for this study has been obtained from the University of Leeds School of Dentistry Research Ethics Committee (DREC).

If you would like to discuss participation in the study or for more information, please contact:

Wendy Thompson will be pleased to talk to you about the study if you have any further questions. She is a dentist and National Institute for Health Research (NIHR) Doctoral Research Fellow (DRF). She can be contacted at dnwt@leeds.ac.uk.

If you have any concerns or if you wish to withdraw from the study, please contact:

Wendy Thompson (dnwt@leeds.ac.uk – NIHR Doctoral Research Fellow)); OR Professor Gail Douglas (0113 343 9214 – PhD supervisor) OR Professor Sue Pavitt (0113 343 6985 - PhD supervisor).

THANK YOU FOR TAKING THE TIME TO READ THIS INFORMATION SHEET
